# Insight Into Novel Anti-tuberculosis Vaccines by Using Immunoinformatics Approaches

**DOI:** 10.3389/fmicb.2022.866873

**Published:** 2022-06-02

**Authors:** Zafran Khan, Daniya Ualiyeva, Obed Boadi Amissah, Sanjeep Sapkota, H. M. Adnan Hameed, Tianyu Zhang

**Affiliations:** ^1^State Key Laboratory of Respiratory Disease, Guangzhou Institutes of Biomedicine and Health, Chinese Academy of Sciences, Guangzhou, China; ^2^University of Chinese Academy of Sciences, Beijing, China; ^3^Guangdong-Hong Kong-Macao Joint Laboratory of Respiratory Infectious Diseases, Guangzhou, China; ^4^China-New Zealand Joint Laboratory on Biomedicine and Health, Guangzhou, China; ^5^Chengdu Institute of Biology, Chinese Academy of Sciences, Chengdu, China; ^6^Faculty of Biology and Biotechnology, Al-Farabi Kazakh National University, Almaty, Kazakhstan

**Keywords:** tuberculosis, *Mycobacterium tuberculosis*, therapeutic vaccine, immunoinformatics, drug resistance

## Abstract

Tuberculosis (TB), an infectious disease, has been a leading cause of morbidity and mortality for decades. The causative agent of TB is the *Mycobacterium tuberculosis* (Mtb) which can infects various parts of the body, mainly the lungs in pulmonary TB cases. *Mycobacterium bovis* Bacillus Calmette–Guerin (BCG) is the only approved vaccine for TB, but its efficiency to combat pulmonary TB is limited. Multidrug-resistant (MDR) TB and extensive drug-resistant (XDR) TB requires the evolution of more potent vaccines. Therefore, this research aims to generate a universal TB subunit vaccine using advanced immunoinformatics techniques. In generating a novel multiepitope subunit vaccine, we selected the conserved and experimentally confirmed antigens Rv0058, Rv0101, and Rv3343. After a rigorous evaluation, the top candidates from predicted Helper T-lymphocytes (HTL), Cytotoxic T-lymphocytes (CTL), and B-cell epitopes were considered potential vaccine candidates. Immunogenicity was enhanced by the addition of an adjuvant to the ultimate construct of the vaccine. B-cell epitopes predictions guaranteed the eventual induction of a humoral response. Thereafter, dynamics simulations and molecular docking validated the vaccine-receptor complex’s stability and high affinity for the immune receptor TLR-3. Also, immune simulations revealed the significantly elevated levels of immunoglobulins such as IgM, cytokines such as interleukin-2, helper T (Th) cells, and cytotoxic T-cell populations. These results agreed with the actual inflammatory response and showed rapid antigen clearance after manifold exposure. Finally, the *E. coli* K12 strain was confirmed *via in-silico* cloning for quality expression. Nevertheless, *in vivo* experiments should be performed to validate the safety of the proposed vaccine and its inherent ability to prevent TB infection.

## Introduction

*Mycobacterium tuberculosis* (Mtb) infects ~10 million individuals each year, and the World Health Organization (WHO) reported that around 1.5 million people died from TB in 2020 (of which 214,000 were HIV positive). Among them, 0.6 million were MDR-TB cases, with an estimated 0.24 million deaths (WHO, 2021). The rapid emergence of MDR-TB strains exacerbates the problem by resulting in irregular and ineffective regimens and minimal TB care. The individuals with low immunity are more vulnerable to TB infection (Singhvi et al., 2020). Thus, their immune systems have inadequate immune responses (Dalsass et al., 2019; Prabowo et al., 2019). The CD4^+^ and CD8^+^ T cells render cellular immune responses by acting against Mtb (Mendez-Samperio, 2016a,b). These T cells, upon stimulation, secrete cytokines triggering an inflammatory response. CD8^+^ cells are responsible for cytotoxicity and cause the lysis of cells that have been infected, thereby causing the removal of TB (Zenteno-Cuevas, 2017).

*Mycobacterium bovis* Bacillus Calmette–Guérin (BCG), the only licensed vaccine against TB that has been used since 1923 showed 0%–80% efficacy rate against pulmonary TB in adolescents and immunization for 10–20 years (Romano and Huygen, 2012; Bali et al., 2015). Nevertheless, the BCG is a live attenuated vaccine. Its use in immunocompromised individuals is related to a possible risk of pathogen restoration to be virulent (Detmer and Glenting, 2006), indicating an unsatisfactory safety rating as an alive-attenuated vaccine. However, there are 16 TB vaccines in various clinical phases, and some of them are live attenuated Mtb vaccines. Previously, low efficacy has been observed of some vaccines based on viral vectors like Crucell-Ad35/AERAS-402 Nad MVA85A (Minch et al., 2015) prior to the vector exposure (Wilkie and Mschane, 2015). Alternatives such as the live prophylactic recombinant vaccines like BCG VPM1002 and MTBVAC (Minch et al., 2015; Mendez-Samperio, 2016a,b) are also available but can revert to pathogenic strains. Thus, immunizing infected persons with subunit-based vaccines reduces the prospect of virulence reversal. Mtb antigens are mixed into the subunit vaccines (M72 and H4). However, they lack immunogenicity and rarely induce long-term immunity, requiring multiple subunit vaccines with adjuvants. Furthermore, the M72/AS01 E in TB-positive and TB-free adults was clinically safe (Gillard et al., 2016). However, some volunteers developed local reactions at injection sites in the course of phase II, which prompted the study’s premature termination. While the peptide vaccines, i.e., H4/IC31, are considered as effective and safe TB vaccines, owing to their efficiency. H4/IC31 generated a robust immunological response in healthy individuals and BCG-vaccinated infants during phase II (Kagina et al., 2014). However, epitopes with almost no reactogenicity (Li et al., 2014) and low production costs (Slingluff, 2011) made it clinically safe and accessible. Various other subunit vaccines consisting of multiple epitopes have been developed as new successful TB vaccines (Ong et al., 2020). In addition to the computational methods, the data from clinical trials have also been used to experimentally evaluate immunological responses by T cells to various Mtb vaccines (Luabeya et al., 2015; Lindestam Arlehamn et al., 2016; Penn-Nicholson et al., 2018; Rodo et al., 2019; Suliman et al., 2019). Rodo et al. inferred that categorizing the vaccines with unique inflammatory response features can provide helpful information to improve the prospect of developing a safe vaccine (Rodo et al., 2019). Thus, the current study aimed to use a reverse vaccinology technique to develop a multi-epitope TB vaccine. A reverse vaccinology technique has recently demonstrated several multiple epitopes vaccines against COVID-19 (Rahman et al., 2020a,b; Kumar et al., 2021), Ebola (Ullah et al., 2020), and Malaria (Damfo et al., 2017). Also, a potential vaccine coding in the Mtb H37Rv genome was created by various immuno-informatics techniques to produce adaptive immunity in numerous B and T cell epitopes (Yazdani et al., 2020). Our findings suggest that the three Mtb proteins (Rv0058, Rv0101, and Rv3343) can be used as multi-epitope vaccine candidates in future laboratory experiments against TB.

The protein, replicative DNA helicase (Rv0058) encoded by the gene *dnaB*, participates in initiation and elongation during chromosome replication and also exhibits DNA-dependent ATPase activity. While the mycobactin synthetase protein B (Rv0101) encoded by the gene *nrp,* is involved in the mycobactin biosynthesis pathway, which is a part of siderophore biosynthesis and plays a key role in lipid metabolism. In addition, the uncharacterized PPE family protein (Rv3343) encoded by the *PPE54*, plays a role in host phagosome maturation arrest.^1^ Thus, we selected the epitopes from the above mentioned Mtb proteins and merged them, to dock with Toll-Like Receptors [TLRs; a group of Pattern Recognition Receptors (PRRs) that bind to the exogenous pathogen-associated molecular patterns (PAMPs) like other PRRs. Hence the main function is to sense the harmfulness and mediate the innate immune response to pathogens (Crozat and Beutler, 2004). TLRs play an important role in innate immune responses to infection] to make a successful, widely available epitope ensemble vaccine that may be utilized to construct a universal vaccine (Watt and Liu, 2020). However, we will validate this vaccine construct *in vivo*, to make it experimentally proven to use against TB.

## Materials and Methods

### Mtb Strain (Antigens) Selection and Protein Sequence Retrieval

**Figure 1:**
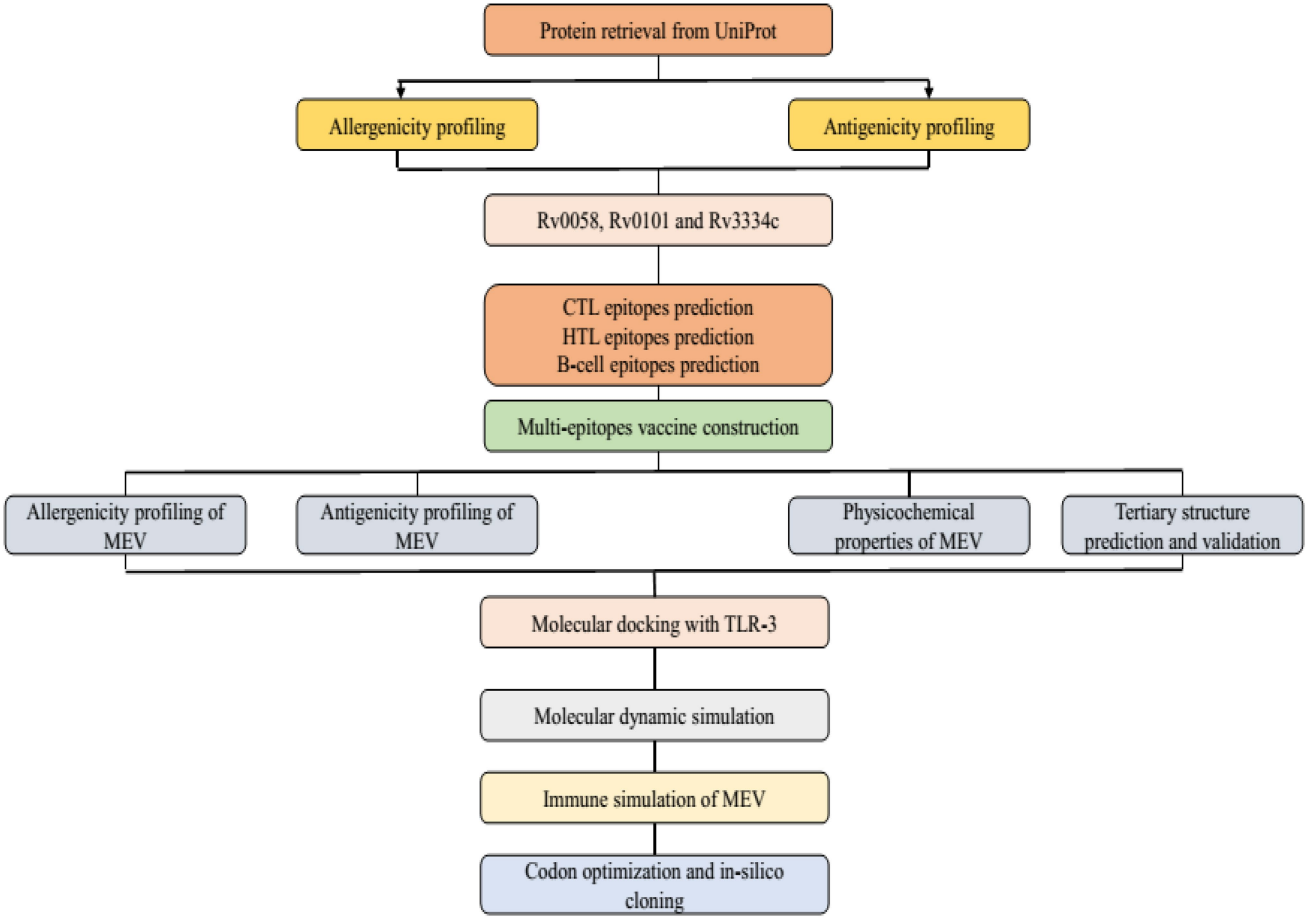
Illustration of study workflow.

Mtb has seven lineages, with lineages 2, 3, and 4 responsible for the typical global spread of TB. Lineage 4 is the most commonly pre-clinically used lineage for vaccine development due to the common H37Rv laboratory strain. However, there is no absolute proof to confirm this selection (Peters et al., 2003). The Uniprot database was used to obtain the Mtb (H37Rv) protein sequences and used for further analysis.^2^ For the specific Mtb epitopes collection, the Immune Epitope Database Analysis Resource (IEDB-AR) was used to identify antigenic proteins.^3^ The three antigenic proteins Rv0058 (Accession No: P9WMR3), Rv0101 (Accession No: Q10896), and Rv3343 (Accession No: Q6MWY2) were selected for this study based on the following qualities: immunogenicity (whether the proteins are immunogenic or not), interferon stimulation, major histocompatibility complex (MHC) molecules binding affinity, and conservancy (Figure 1).

### Cytotoxic T Lymphocytes Epitopes Prediction

Identification of the cytotoxic T lymphocytes (CTL) epitope is a critical step in developing a multiepitope subunit vaccine (MSV). To predict CTL epitopes, the protein sequences were analyzed with NetCTL 1.2 server.^4^ Predictions were made using the MHC class I binding peptide weight matrix, transporter associated with antigen processing (TAP) and, proteasomal C-terminal cleavage score. A threshold of 0.75 was established for the prediction of epitopes. The combined score was used to categorize the predicted epitopes. However, the server-allowed CTL epitopes prediction is restricted to 12 MHC-1 supertypes, though this study focuses on the A1 supertype (Peters et al., 2003).

### Helper T Lymphocytes Epitopes Prediction

IEDB’s server was used to predict the helper T lymphocytes (HTL) epitopes (Zhang et al., 2008). The 7-allele human leukocyte antigen (HLA) was used as a reference set to predict the epitopes, and HLA-DR was selected for *Homo sapien*. Afterward, a 15-mer length epitope with a percentile rank classification was obtained. Next, comparing peptides to several 15-mer nucleotide databases on SWISSPROT, with a percentile assigned to them, those with the lowest rank have a high affinity for MHC-II.

### Interferon-Gamma Inducing Epitopes Prediction

The 15-mer was submitted to the interferon-gamma (IFN-γ) epitope server to speculate whether the HTL epitopes can induce the IFN-γ immune response.^5^ The epitopes of the IFN-γ were predicted based on overlapping sequence constructs. The model was anticipated by comparing IFN-γ and non-IFN-γ producing cells (Dhanda et al., 2013) using a Support Vector Machine (SVM) prediction system. Thus, the high-response producing IFN-γ epitopes were selected for the final vaccine.

### Linear B-Cell Epitopes Prediction

Epitopes of B-cells are also critical in developing vaccines that modulate antibody-mediated immunity. Linear B-cell epitope prediction was made with the ABCpred server.^6^ The 16-mer epitopes were predicted, and an overlapping filter value of 0.51 threshold was kept on using a neural network. The epitope with the highest score of 0.9 was chosen for vaccine development.

### Protein Sequences Antigenicity Prediction

The ability to be antigenic is a crucial feature of the vaccine. The antigenicity of selected genes was assessed with a 0.4 threshold using VaxiJen 2.0 server.^7^ For vaccine development, potential antigen epitopes were selected. The vaccine’s antigenicity was estimated using the auto and cross-covariance (ACC) transformation with essential amino acid (aa) characteristics into uniform vectors. VaxiJen uses an algorithm that analyses protein physicochemical properties to determine whether they are antigenic or not (Pandey et al., 2018).

### Protein Sequences Allergenicity and Toxicity Prediction

Identifying allergens is a crucial step in developing a vaccine. Using the SVM module and a threshold of −0.4, the Algpred server checked the allergenic properties of protein sequences.^8^ Protein sequences with non-allergenic properties were chosen for further research. Finally, the ToxinPred server was used to verify all of the epitopes for toxic and non-toxic epitopes (Gupta et al., 2013).^9^ These characteristics have also confirmed the vaccine’s overall design.

### Multi-epitope Subunit Vaccine Sequence Construction

For the final Multi-epitope subunit vaccine (MSV), highly antigenic, non-allergenic, immunogenic, and non-toxic epitopes were chosen. The CTL epitopes were combined by an AAY linker, whereas HTL and B-cell epitopes had a GPGPG linker. A griselimycin (APD ID: AP02688) adjuvant was added through an EAAAK linker to boost the vaccine’s immunogenicity (Kling et al., 2015). The griselimycin sequence was accessed on the Antimicrobial Peptide Database (APD).^10^

### Prediction of Physicochemical Characteristics and Solubility

The vaccine construct was subjected to Expasy Protparam^11^ to determine the physicochemical characteristics such as the aliphatic index, molecular weight (MW), theoretical isoelectric point (pI; Hennebert et al., 2015) and other necessary information. The Protein-Sol server^12^ determined the solubility value of the MSV. A scaled solubility value of more than 0.45 indicates that the protein is more soluble than that of the average soluble *E. coli* protein in the experimental solubility dataset (Hebditch et al., 2017). Proteins of lesser scaled solubility value are expected to be insoluble.

### Secondary Structure Prediction

The PSIPRED and SOPMA are two online servers used to construct the secondary structure of the designed vaccine. PRISPRED^13^ predicts transmembrane helix, transmembrane topology, domains, folds, and other information (Mcguffin et al., 2000). The SOPMA server^14^ determined the additional characteristics of MSV (Combet et al., 2000).

### Tertiary Structure Prediction

Robetta server was used to predict the tertiary structure of the designed vaccine^15^ (Kim et al., 2004) and provide highly accurate results. Robetta offers both *ab initio* and relative protein domain models. The structures detected and aligned by HHSEARCH, SPARKS, and Raptor were used to create relative models. Loop domains were extracted from the fragments and were optimized to fit the aligned template structures. The Rosetta *de novo* protocol is used to create *de novo* models. If there is no template, the *de novo* Rosetta fragment insertion method was preferred.

### Tertiary Structure Refinement

The GalaxyRefne web server^16^ was used to fine-tune the MSV 3D model. GalaxyRefne server is premised on a sophisticated routine that accomplishes reassembling and uses molecular dynamics modulation for structural refinement. It improves the models provided by protein structural predictors by improving global and local structure quality (Heo et al., 2013). Also, the energy was minimized using the Chiron server.^17^

### Validation of Tertiary Structure

Validation of the tertiary structure is important in vaccine development because it reveals possible flaws in the anticipated model (Khatoon et al., 2017). The ProSA-webserver was used to evaluate the total quality score of the precise input structure for 3D structure validation.^18^ The structural design is likely to contain errors should Z scores be outside the range of native protein (Wiederstein and Sippl, 2007). To evaluate unbonded inter-atomic interactions, high-resolution crystallography structures were predicted using ERRAT.^19^ A Ramachandran plot, which shows the number of residues in either allowed or disallowed domains by describing the consistency of the structure (Lovell et al., 2003), was assessed and retrieved from RAMPAGE.^20^

### Discontinuous B-Cell Epitope Prediction

The discontinuous epitopes in the 3D structure were predicted with ElliPro, and 80% of evaluated B cell epitopes were discontinuous.^21^ ElliPro calculates the adjacent cluster residues using the protrusion index (PI). The average PI value of the epitope’s residues is used to calculate a score for each output epitope. Protein residues make up 90% of an ellipsoid with PI values of 0.9. The PI value of each residue was calculated using the midpoint mass outside the major ellipsoid.

### Vaccine Molecular Docking With an Immune Receptor

Molecular docking is based on the contact of a particular immune receptor with an antigenic molecule, which results in an effective immune response. The Protein Databank (PDB)^22^ was used to obtain Toll-Like Receptor 3 (TLR3; PDB ID: 5GS0). The docking was performed by HawkDock^23^ and FireDock,^24^ which refine the contact between the antigen and the receptor (Bhati et al., 2018). The HawkDock server was used to estimate the Molecular Mechanics/Generalized Born Surface Area (MM-GBSA) for affinity prediction (Weng et al., 2019). The lowest predicted score is the preferred score.

### Normal Mode Analysis

Simulation-based analysis of vaccine structures was conducted with the most significant findings on docking. The iMODS web-server^25^ facsimiled the dynamic analysis, with a defined and estimated protein flexibility (Lopez-Blanco et al., 2011).

### Codon Optimization

The route to optimizing codon increased the expression of recombinant protein. The codon adaptation index (CAI) and the GC contents were utilized to determine the protein expression levels in the K12 *E.coli* strain (most frequently used bacterial hosts for the production of recombinant proteins on an industrial scale) *via* Java Codon Adaptation Tool (JCat).^26^ The optimum CAI score ranges from 0.8 to 1.0, with an optimum GC content of 30% to 70% (Abraham et al., 2021). A recombinant DNA was designed between the optimized gene sequence of the vaccine and pET-21(+) expression vector at *Ahd*I and *Sty*I restriction sites and transformed in *E. coli*. The transformed clones were sequenced to select the correct clone containing the functional vaccine gene.

### Immune Simulation

The inflammatory response characteristics of the modeled vaccine went through *in silico* documentation using C-ImmSim.^27^ C-ImmSim identified mammalian adaptive immune responses to a vaccine construct. The prophylactic TB vaccination was injected in three doses within 4 weeks intervals. Simulation parameters included 1, 84, and 168-time intervals. The simulation volume was set to 50 and the simulation steps to 1,000, respectively (with LPS-free vaccine injection, random seed = 12,345).

## Results

### Protein Sequences Retrieval

Mtb (H37Rv) amino acid sequences of Rv0058, Rv0101, and Rv3343 proteins were obtained from Uniprot KB as FASTA files and used for B-cell and T-cell epitopes prediction for the development of the MSV against TB.

### Prediction of CTL-Epitope

The epitopes of the three nominated proteins, Rv0058 (13 epitopes), Rv0101 (42 epitopes), and Rv3343 (24 epitopes), were predicted with the threshold set to the value of epitopes documentation *via* NetCTL 1.2 web-server. Among them, the seven predicted CTL epitopes were finally selected for vaccine development based on strong ratings for binding affinity to MHC class I and other immunological parameters, as shown in Table 1.

**Table 1:** Selected CTL epitopes that met all of the criteria for antigenicity, non-allergenicity, and non-toxicity, and the ability to bind efficiently to MHC-I A1-supertype alleles.

Protein	Peptide sequence	MHC binding affinity	Rescale binding affinity	C-terminal cleavage affinity	Transport efficiency	Prediction score
Rv0058	LSDMRSGRM	0.2786	1.1827	0.5806	0.2740	1.2835
	PTNGQGRVY	0.2027	0.8608	0.7749	2.1900	1.0866
Rv0101	LTADLSAAY	0.7063	2.9987	0.9596	2.7790	3.2816
	VSAPTIINY	0.5144	2.1839	0.9199	3.0210	2.4730
Rv3343	FSIPVTFSY	0.4237	1.7988	0.9743	2.9090	2.0904
	VSESIPLNF	0.2736	1.1617	0.8822	2.5030	1.4192
	YSTPALTLF	0.2062	0.8754	0.8222	2.5730	1.1274

### Prediction of HTL-Epitope

The HTL epitopes were identified *via* IEDB website as high binding MHC class II epitopes for HLA-DR. Eight epitopes were selected based on their immunological characteristics, as shown in Table 2. HLA-DRB5*01:01(264–278), HLA-DR B5*01:01(31–45), HLA-DRB3*01:01(45–59), HLA-DRB1*03:01 (823–837), HLA-DRB3*01:01(60–74), HLA-DRB5*01:01(460–474), HLA-DRB5*01:01 (368–382), HLA-DRB5*(548–562).

**Table 2:** HTL epitopes that met all of the criteria for antigenicity, nonallergenicity, and nontoxicity, as well as the ability to induce an IFN-γ immune response.

Protein	Allele	Start	End	Peptide sequence	Percentile rank	Method	IFN-γ result
Rv0058	HLA-DRB5*01:01	264	278	VMRLLSAEAKIKLSD	0.43	SVM	Positive
	HLA-DRB5*01:01	31	45	GRKEVFRLRLASGRE	0.66	SVM	Positive
	HLA-DRB3*01:01	45	59	TRILRADTGAEVAFG	1.4	SVM	Positive
Rv0101	HLA-DRB1*03:01	823	837	DQRGASLVVDWPASV	1.20	SVM	Positive
	HLA-DRB3*01:01	60	74	AALFVLDSWLRPVPA	2.20	SVM	Positive
	HLA-DRB4*01:01	460	474	TRIRLVLVSLGVSSF	2.70	SVM	Positive
Rv3343	HLA-DRB5*01:01	368	382	LGLTVRYLTPHSKWS	0.08	SVM	Positive
	HLA-DRB1*07:01	548	562	HSDVMYRSVLALLML	1.40	SVM	Positive

The lowest the percentile rank the higher binding affinity toward the MHC-II. Individual epitope percentile score reflects its ability of binding to MHC-II.

### Prediction of IFN-γ Inducing Epitopes

IFN-γ is a cytokine produced by CTL and natural killer cells that help pathogen evasion within the cell. The SVM route identified the epitopes that induce IFN-γ. Four IFN-positive HTL epitopes have been selected for the development of the vaccine.

### Prediction of Linear B-Cells Epitopes

ABCpred’s website anticipated the epitopes of the B cell. Table 3 enlists all prophesied epitopes of more than a 0.9 affinity score.

**Table 3:** Linear B cell epitopes with a binding score of greater than 0.9 chosen for the final vaccine.

Protein	Peptide sequence	Start position	Predicted score
Rv0058	MMDIQLHEPTMWKHSP	739	0.90
Rv0101	CAAISAPLRPGSGMPP	1777	0.98
Rv3343	RGDYQGLLGFSSGANV	423	0.94

### Construction of MSV

**Figure 2:**
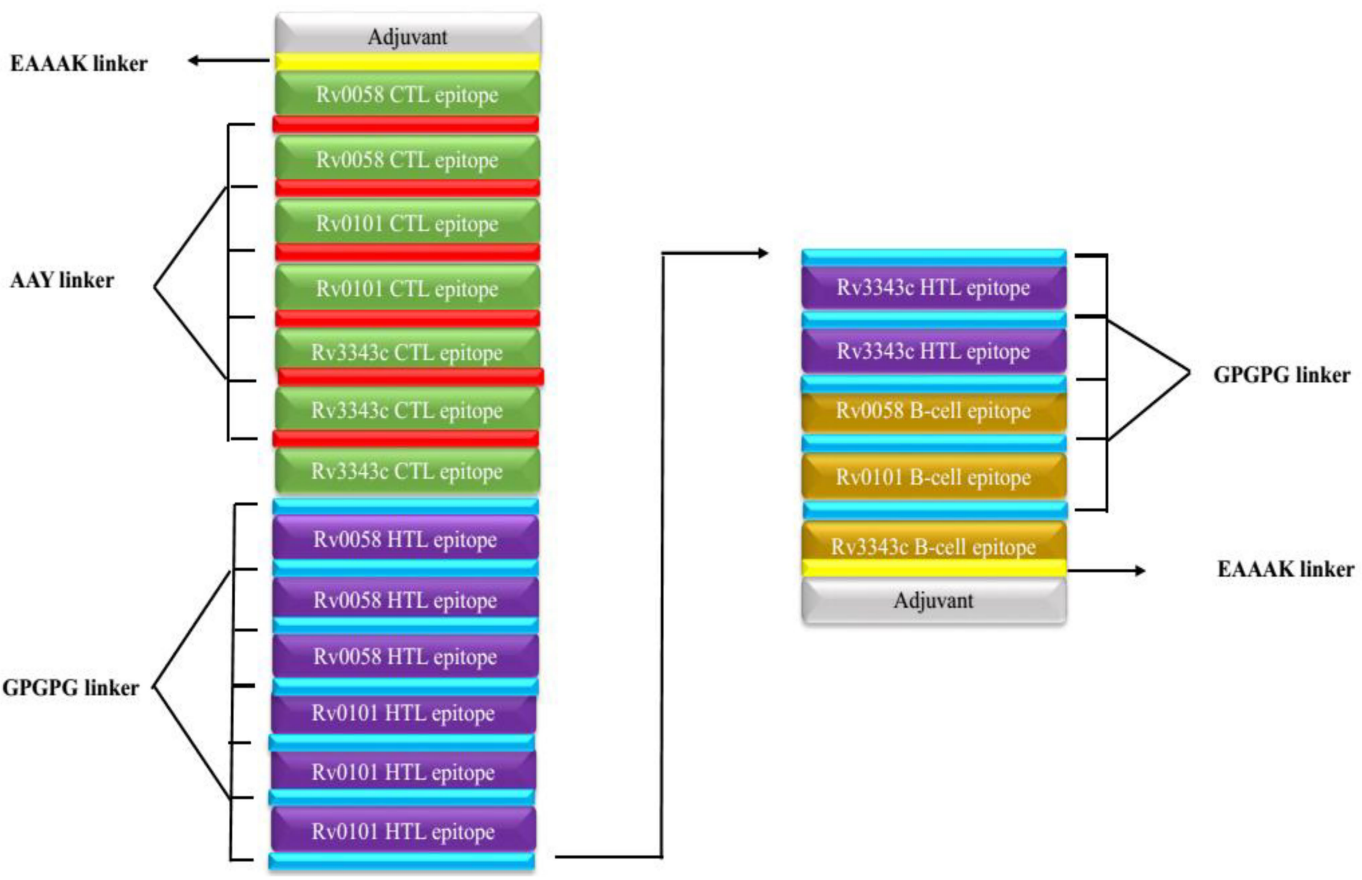
Schematically presentation of final MSV. An EAAAK linker (yellow) was used to connect the 309-amino-acid long peptide sequence with adjuvant (grey) at both the N and C terminals to the multiepitope sequence.GPGPG linkers (blue) are used to connect B cell and HTL epitopes, while AAY linkers (red) are used to connect CTL epitopes.

Seven CTL, eight HTL, and three epitopes of B cells were selected after meeting the affinity, toxicity, and allergenicity requirements. Griselimycin adjuvant (APD ID: AP02688) was added to both terminals of the vaccine to enhance immunogenicity. Adjuvant epitopes were linked with EAAAK linkers, CTL and HTL epitopes had an AAY linker, and B-cell epitopes had a GPGPG linker. The vaccine sequence was double-checked. The final multi-epitope peptide of the vaccine of this research is shown schematically in Figure 2.

### Prediction of Antigenicity and Allergenicity Vaccine Candidates

VaxiJen v2.0 server was used to evaluate the antigenicity of the three selected proteins. The result indicated a high antigenic potency of all three chosen proteins. The antigenicity criterion in the VaxiJen method was standardized to a value of 0.4. The antigens Rv0058, Rv0101, and Rv3343 had antigenicity scores of 0.46, 0.48, and 0.62. The server predicted the vaccine’s antigenicity to the value of 0.86. According to our findings, the current vaccine possesses high antigenic properties. The Algpred servers also indicated a non-allergenic vaccine series.

### Prediction of the Physicochemical Properties and Solubility

**Figure 3:**
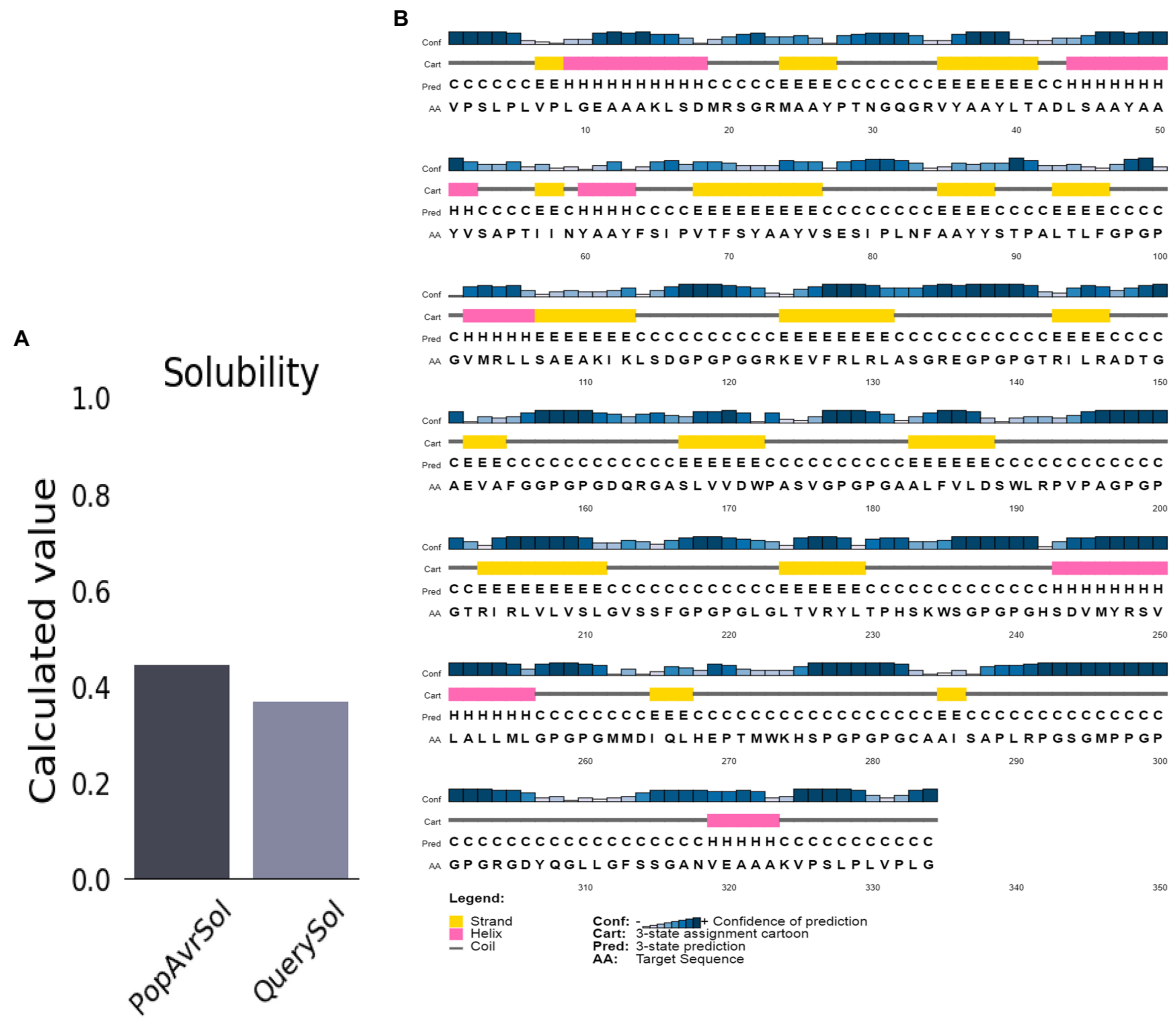
Predicted vaccine construct’s solubility and secondary structure. **(A)** Solubility analysis of vaccine constructs with a score of 0.557 after expression, by using ProtSol. **(B)** Secondary structure prediction of vaccine constructs with (20.0%) alpha-helices, (21.0%) beta-strands, and (58.0%) coils, by using the PSIPRED server.

The molecular weight and the protein’s pI were 34.1 kDa and 9.5, respectively. The half-life of mammalian reticulocytes, yeast, and *E. coli in vitro* was 100, >20, and >10 h, respectively. The protein’s instability index (II) was 29.94, indicating high stability (Instability II > 40). A high aliphatic index score of 84.19 (Ikai, 1980) suggests the vaccine is thermostable. The vaccine structures’ Grand Average of Hydropathicity (GRAVY) of 0.102 indicates a hydrophilic vaccine. The antigen had a solubility score of 0.371, indicating particulate/aggregated antigen (Figure 3A).

### Prediction of Secondary Structure

The structure of the vaccine upon sequences analysis indicated a 20.66% α-helix, 24.85% β-strand, 50% coil in total (Figure 3B).

### Modeling of Tertiary Structures

**Figure 4:**
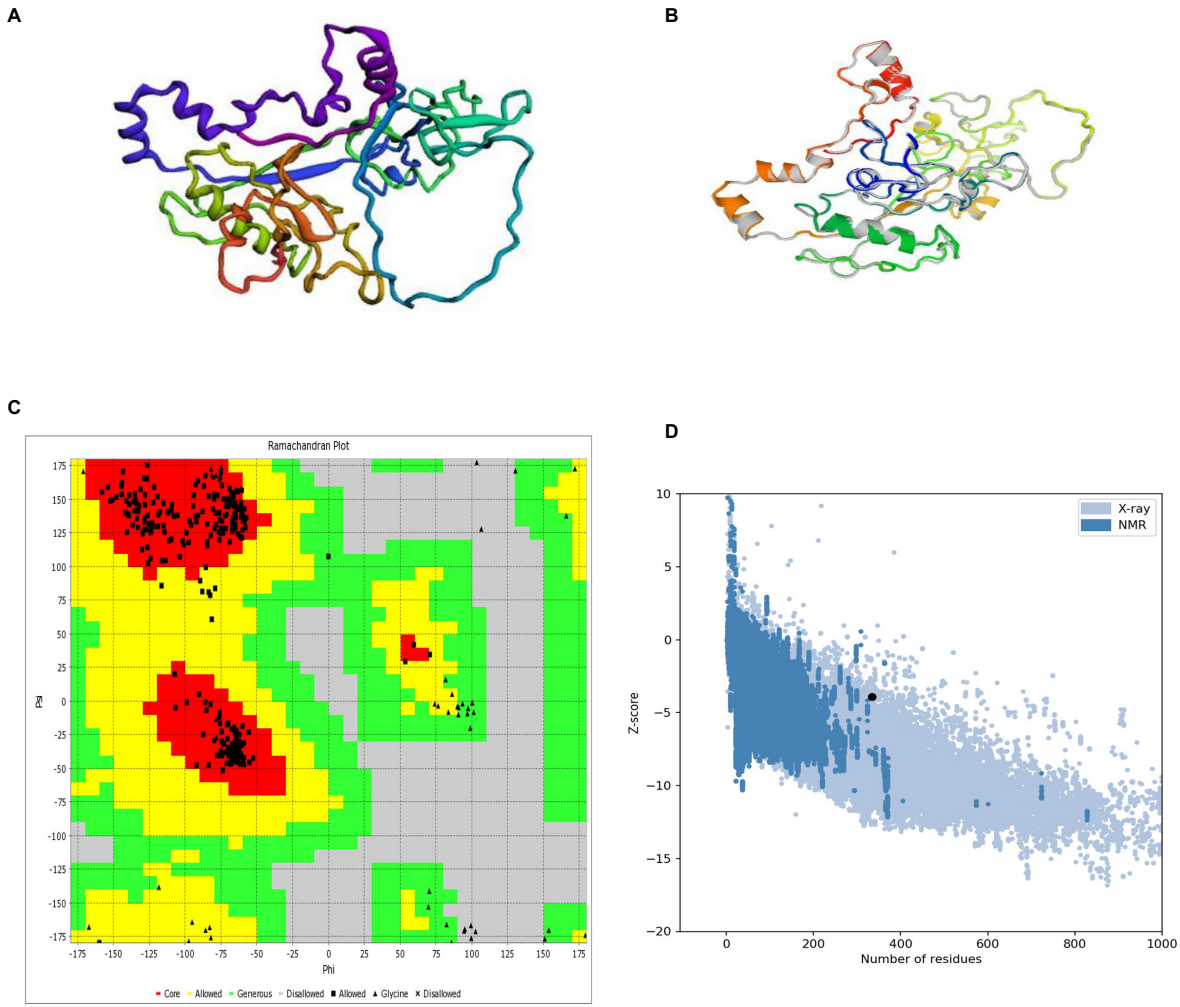
3D modeling, refinement, and validation of proteins. **(A)** Following homology modeling, the I-TASSER server produced a 3D model of a MSV. **(B)** Refinement: superimposition of a refined 3D structure (colored) on a “crude model” (gray) by the GalaxyRefne server. **(C)** Validation: Ramachandran plot analysis of protein residues, showing 85.9% in favored, 8.9% in allowed, and 5.2% in disallowed regions; **(D)** ProSA-web, with a Z score of 1.39.

The Robetta server predicted five 3D models of the vaccine, with confidence score (C-score) values (0.12). Robetta estimated a candidate’s 3D model using a comparative modeling technique. The C score ranges from −5 to 2. A score of 2 indicates extreme confidence. Finally, the best model structure with a C value of 0.12 was chosen for further investigation (Figure 4A). The structure’s root means square deviation (RMSD) was 0.226 Å.

### Refinement of the Tertiary Structure

GalaxyRefne website was employed to improve the quality of the protein. The Chiron server performed loop refinement and energy minimization on the protein structure. On the GalaxyRefne web-server, the original “crude” vaccine model was refined into five models. The fifth model showed superiority over the other developed forms based on variables such as 0.9648 GDT-HA, 0.383 RMSD, and 1.606 MolProbity. Also, the fifth model had a clash value of 12.4 with a low rotamers value of 0.4, and a Rama preferred value of 99.7. Thereby model 5 was chosen as the subject of further investigation (Figure 4B).

### Validation of the Tertiary Structure

The RAMPAGE server exposed the improved structure to a Ramachandran plot analysis. The Procheck and VADAR web server plot revealed 95.49% residues in preferred domains and 4.6% in allowed domains (Figure 4C). The crude 3D model was checked for accuracy and possible errors by the ProSA-web and ERRAT. ERRAT refinement indicated a general quality of 91.91%. Also, the Z score assessment of the vaccine input by the ProSA server was −3.93 (Figure 4D). The unique content of the 3D modeled protein has been confirmed by the results of these structure validation servers.

### Conformational B-Cell Epitopes Prediction

**Figure 5:**
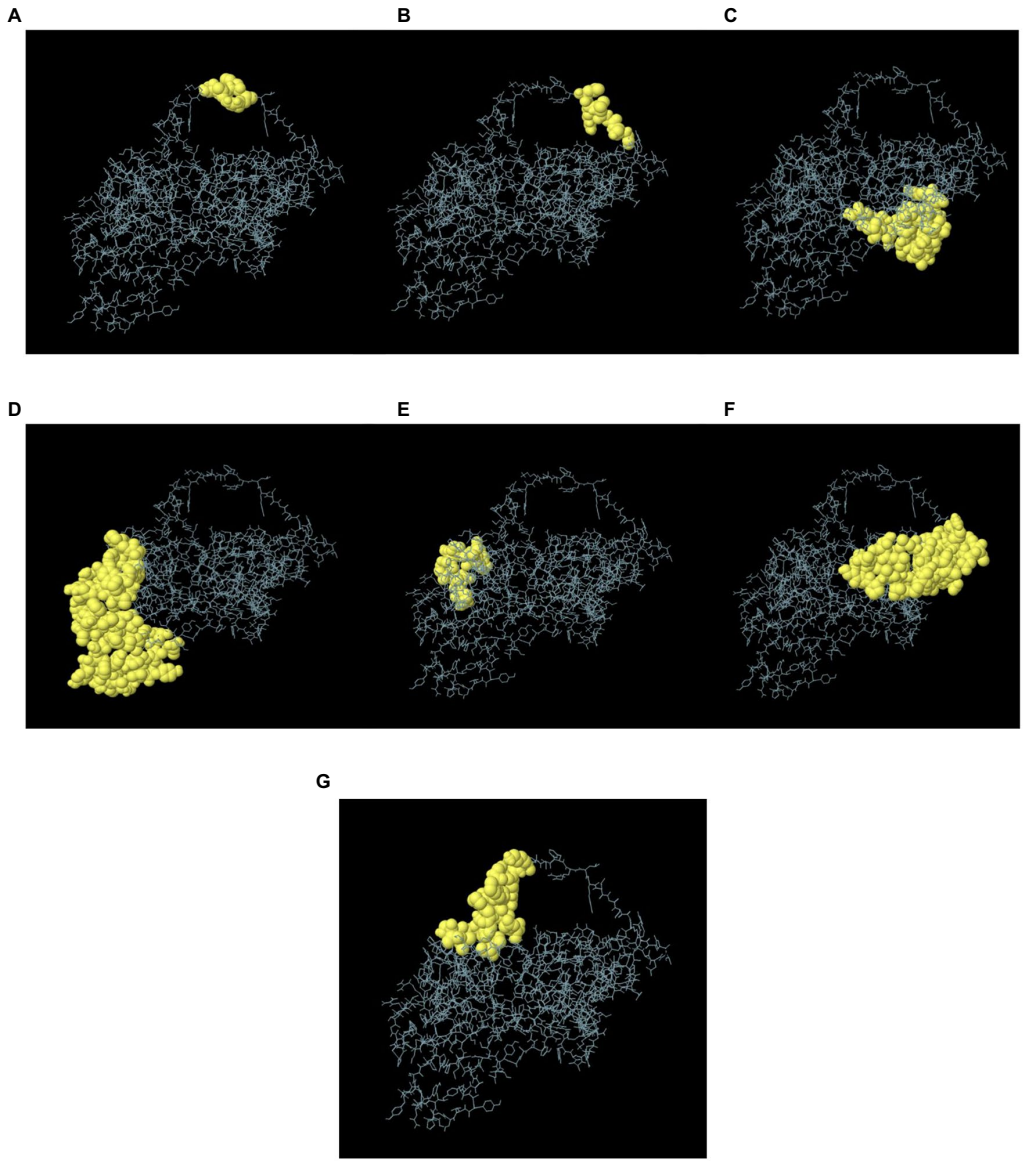
A 3D representation of the designed MSV conformational or discontinuous B cell epitopes. **(A–G)** The conformational or discontinuous B cell epitopes are represented by yellow surfaces, while grey sticks represent the bulk of the polyprotein.

**Figure 6:**
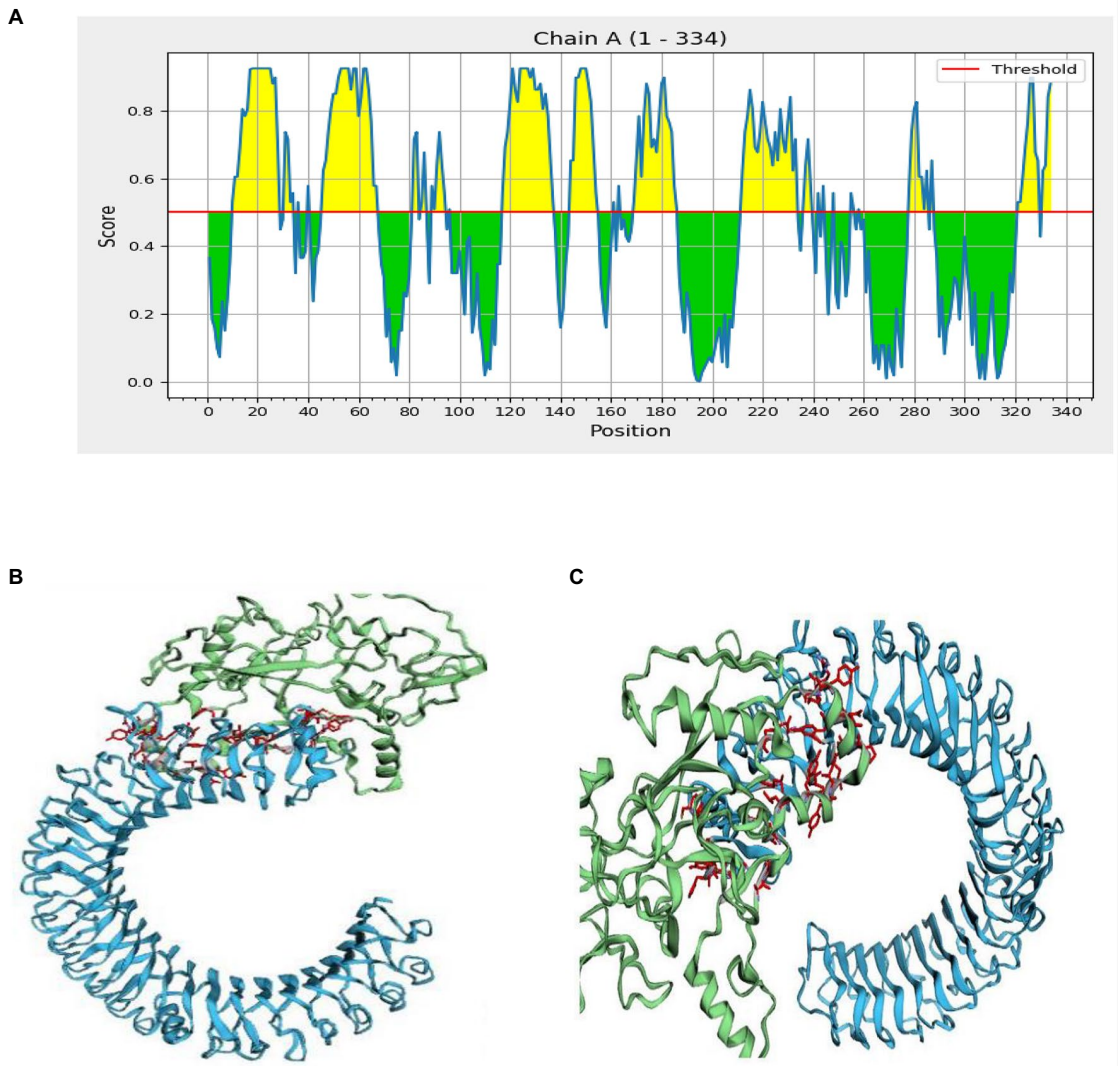
Interaction of the ligand protein (MSV) and the receptor protein in discontinuous B cell epitopes (TLR-3). **(A)** In the MSV, the individual predicted score of discontinuous B cell epitopes. **(B,C)** The ligand protein is highlighted in green, while the receptor protein is highlighted in blue.

In the seven discontinuous epitopes of the B cells, the values of the 192 residues found ranged from 0.628 to 0.918. Conformational epitopes had a size range of 4–64 residues. The value ≥ 0.628 was chosen for discontinuous peptides (Figures 5A–G; Table 4). Several discontinuous epitope residues were anticipated from vaccine sequence lengths of 125–128 (4 epitope residues), 9–28, 31–35, 164–165, 301–302 (6 epitope residues), 40, 43, 46–69, 212–232, 235–242, 244, 248, 255–260 (64 epitope residues), 318, 321–334 (15 epitope residues), 78–86, 88–97, 135–138, 144–155. Figure 6A indicates the score of each discontinuous epitope.

**Table 4:** Conformational B cell epitopes residues of the designed MSV, predicted by ElliPro.

No.	Residues	No. of residues	Score
1	A:E125, A:V126, A:F127, A:R128	4	0.918
2	A:L129, A:R130, A:L131, A:A132, A:S133, A:G134	6	0.864
3	A:L9, A:G10, A:E11, A:A12, A:A13, A:A14, A:K15, A:L16, A:S17, A:D18, A:M19, A:R20, A:S21, A:G22, A:R23, A:M24, A:A25, A:A26, A:Y27, A:P28, A:G31, A:Q32, A:G33, A:R34, A:V35, A:R164, A:G165, A:G301, A:P302	29	0.701
4	A:L40, A:D43, A:A46, A:A47, A:Y48, A:A49, A:A50, A:Y51, A:V52, A:S53, A:A54, A:P55, A:T56, A:I57, A:I58, A:N59, A:Y60, A:A61, A:A62, A:Y63, A:F64, A:S65, A:I66, A:P67, A:V68, A:T69, A:G212, A:V213, A:S214, A:S215, A:F216, A:G217, A:P218, A:G219, A:P220, A:G221, A:L222, A:G223, A:L224, A:T225, A:V226, A:R227, A:Y228, A:L229, A:T230, A:P231, A:H232, A:W235, A:S236, A:G237, A:P238, A:G239, A:P240, A:G241, A:H242, A:D244, A:R248, A:M255, A:L256, A:G257, A:P258, A:G259, A:P260, A:M262	64	0.685
5	A:N318, A:A321, A:A322, A:A323, A:K324, A:V325, A:P326, A:S327, A:L328, A:P329, A:L330, A:V331, A:P332, A:L333, A:G334	15	0.665
6	A:S79, A:I80, A:P81, A:L82, A:N83, A:A85, A:A86, A:Y88, A:S89, A:T90, A:P91, A:A92, A:L93, A:T94, A:L95, A:F96, A:G97, A:R135, A:E136, A:G137, A:P138, A:I144, A:L145, A:R146, A:A147, A:D148, A:T149, A:G150, A:A151, A:E152, A:V153, A:A154, A:F155, A:S167, A:L168, A:V169, A:V170, A:D171, A:W172, A:P173, A:A174, A:S175, A:V176, A:G177, A:P178, A:G179, A:P180, A:G181, A:A182, A:A183, A:L184	51	0.657
7	A:S115, A:D116, A:G117, A:P118, A:G119, A:P120, A:G121, A:G122, A:R123, A:K124, A:P277, A:G278, A:P279, A:G280, A:P281, A:G282, A:C283, A:A284, A:A285, A:I286, A:S287, A:A288, A:L310	23	0.628

### Molecular Docking

Inter protein docking was performed with the PatchDock, and HawkDock servers in an attempt to enhance the predicted efficiency of the vaccine. Further, the FireDock server investigated the docked complexes generated by PatchDock tools in more detail (Figure 6B). The global energy of docked complexes was predicted using FireDock, and HawkDock derived the ranking free-binding energy (kcal/mol). The TLR-3 complex-integrated vaccine had an excellent free-binding energy score of 35.88 kcal/mol, according to the PatchDock server. However, the best performance of the vaccine, according to the MM-GBSA analysis, revealed free-binding energy of −63.08 kcal/mol as shown in the HawkDock server’s nominated TLR-3 in Figure 6C.

### Normal Mode Analysis

**Figure 7:**
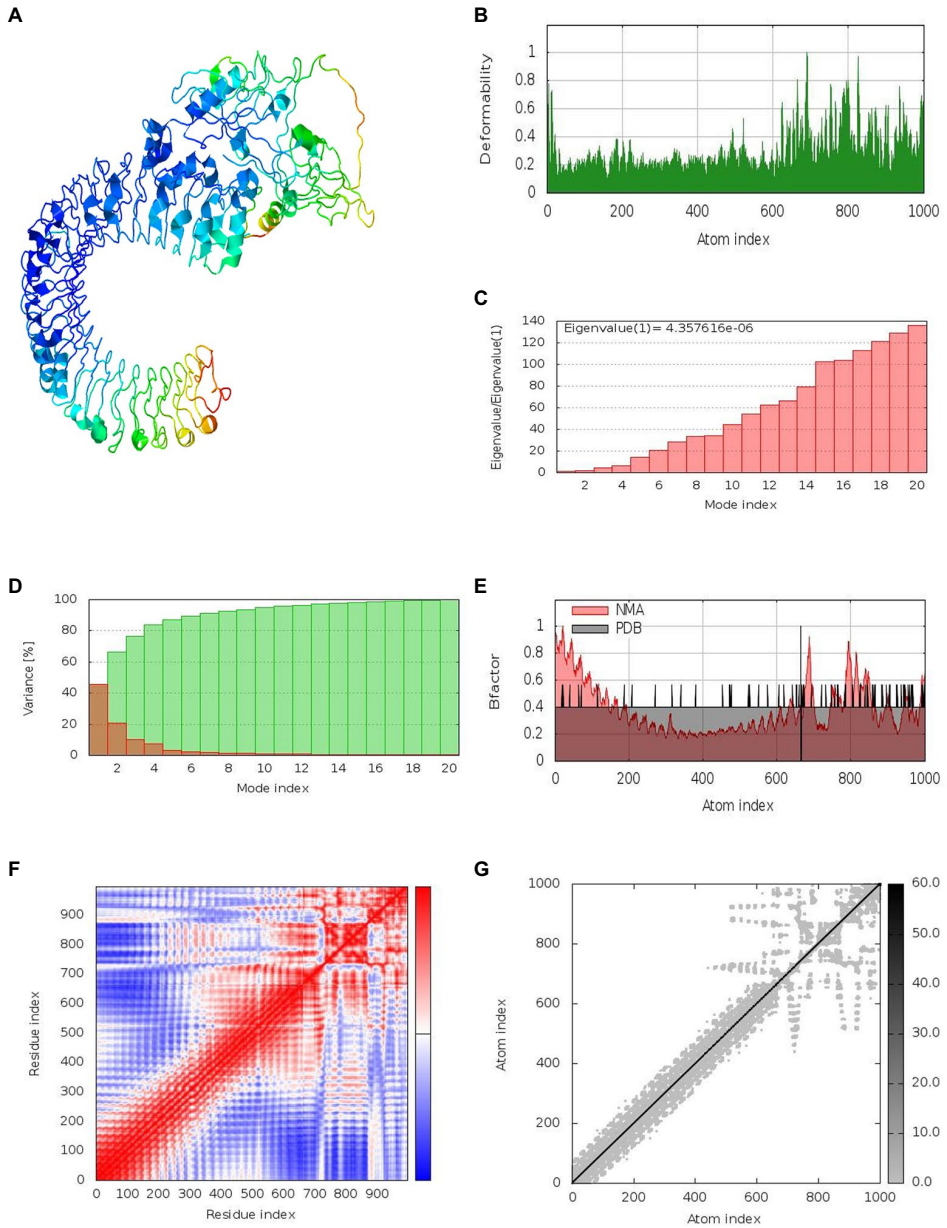
Results of normal mode analysis of a vaccine construct docked with TLR-3. **(A)** NMA mobility, **(B)** deformability, **(C)** eigenvalues, **(D)** variance (individual variances shown in red color and green color indicates cumulative variances), **(E)** Bfactor, **(F)** co-variance map (correlated (red), uncorrelated (white) or anti-correlated (blue) motions), and **(G)** elastic network (darker gray regions indicate stiffer regions).

Together with the docked TLR-3 complex, the models were subjected to normal mode analysis (NMA) (Figure 7A). The goal of the simulation study was to determine the stability and mobility of atoms and molecules in the vaccine construct. The peak in the deformability represents the protein’s deformable domains (Figure 7B). The complex’s eigenvalue was 4.357616e-06 (Figure 7C). The cumulative variance is green on the variance graph, while the individual variance is red (Figure 7D). The relationship of the docked complex to the NMA and PDB sectors is depicted in the B-factor graph (Figure 7E). In the complex’s co-variance map, red indicates correlated motion between two residues, white indicates uncorrelated motion, and blue indicates anti-correlated motion (Figure 7F). Atoms with dark gray regions depicted stiffer domains on the elastic map (Figure 7G).

### *In silico* Cloning and Codon Optimization

**Figure 8:**
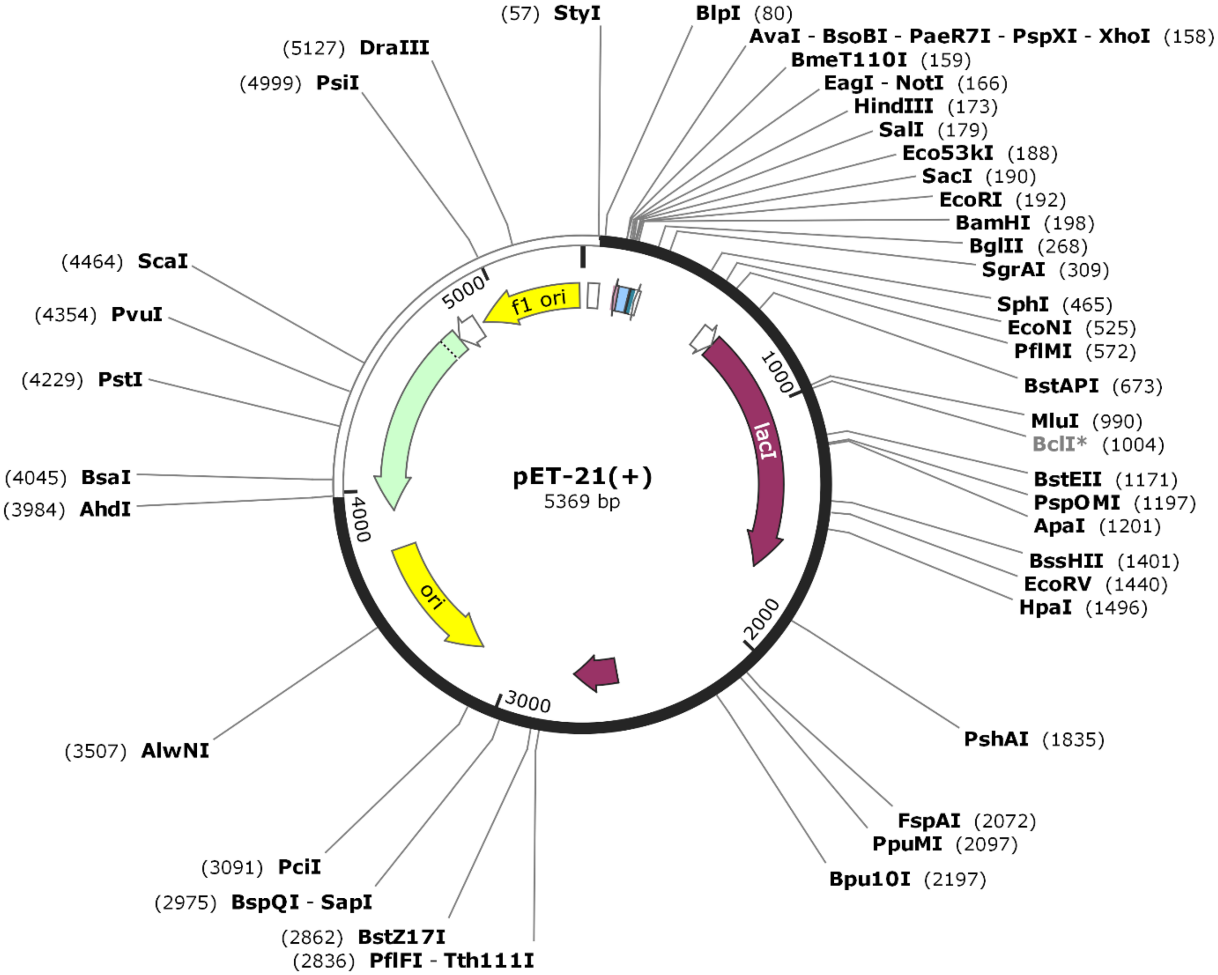
The sequence of the vaccine was optimized and inserted into *E.coli* vector pET-21(+) (57), between AhdI (3984) and StyI (57). The white color represents the inserted DNA sequence.

Codon optimization was performed to enhance protein expression in *E. coli* using Java Codon Adaptation Tool (JCat). The predicted CAI value of 0.968 with an average GC content of 59.48% indicated high expression *in E. coli*. The recombinant pET-21(+) vector was generated with SnapGene software (Figure 8).

### Immune Stimulation

**Figure 9:**
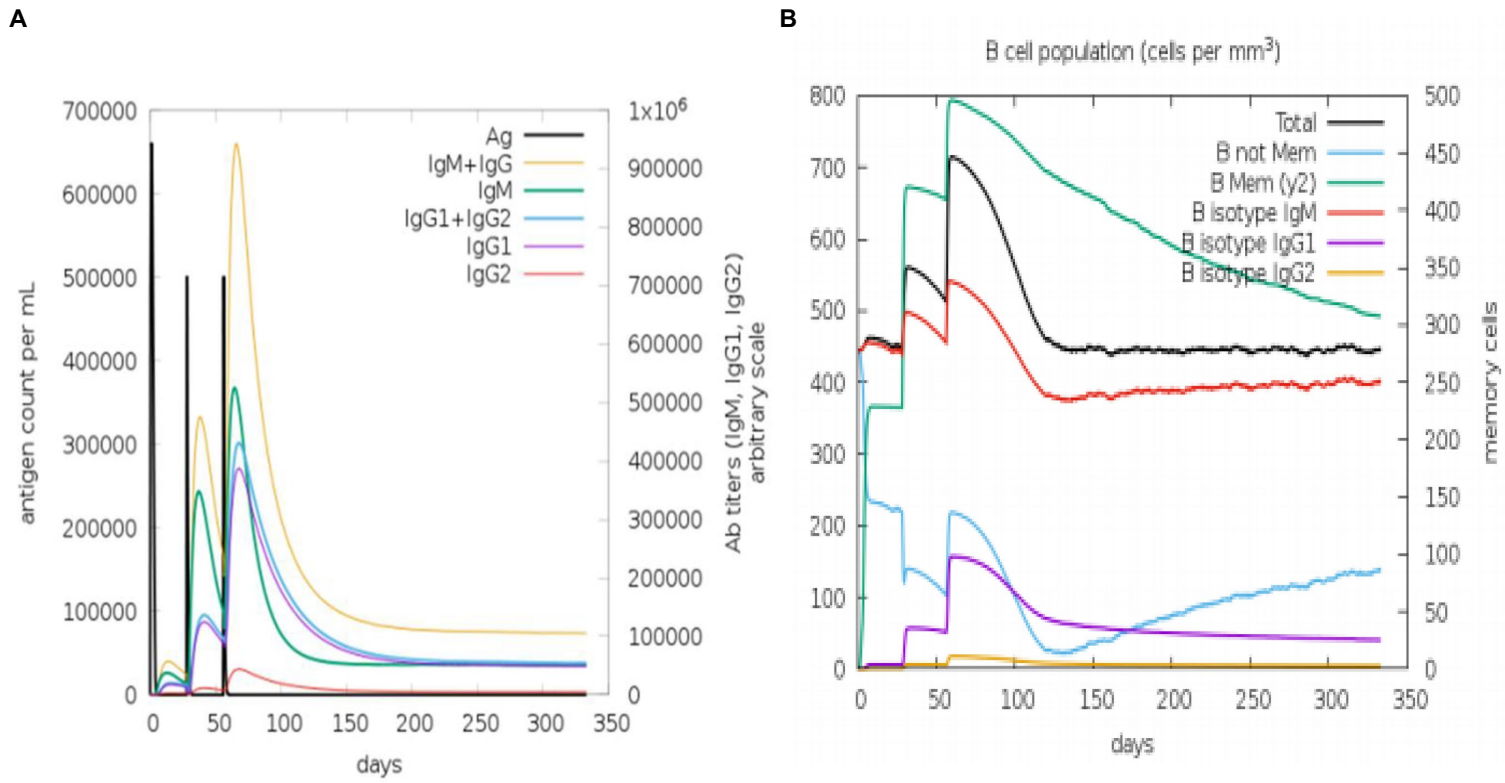
An *in silico* immune simulation with the construct is presented by C-ImmSim. **(A)** Immunoglobulin production in response to antigen injections (black vertical lines); colored peaks indicate specific subclasses. **(B)** After three injections, the evolution of B-cell populations.

**Figure 10:**
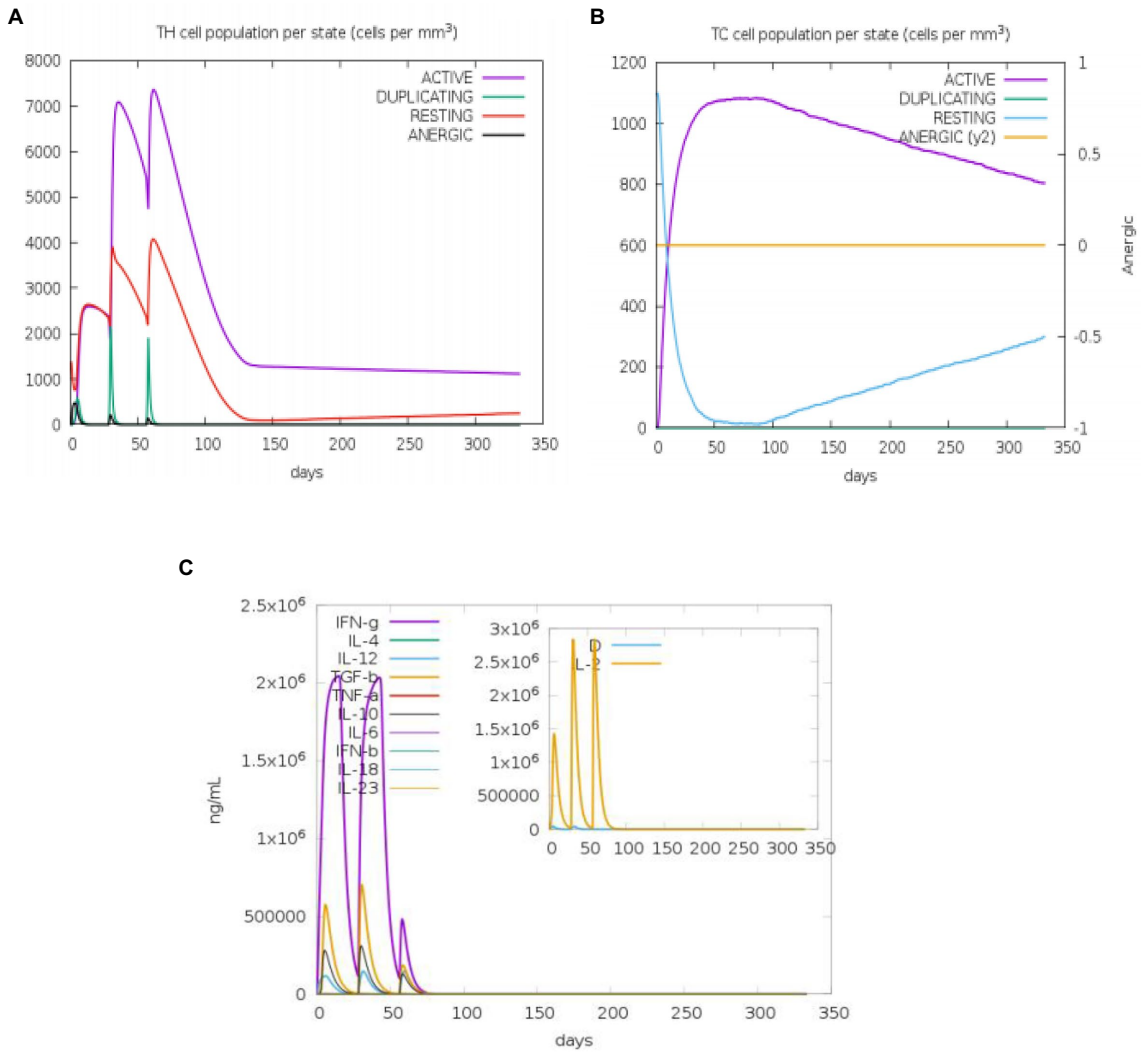
An *in silico* immune simulation with the construct is presented by C-ImmSim. After the injections, **(A)** T-helper cell populations per state evolved, and **(B)** T-cytotoxic cell populations per state evolved. The resting state describes cells that have not been exposed to the antigen, whereas the anergic state describes T-cell tolerance to the antigen as a result of repeated exposures. **(C)** The cytokine levels after injections are shown in the main plot. The Simpson index, D, is shown by the dotted line in the insert plot of IL-2 levels. D is a diversity metric. The emergence of different epitope-specific dominant T-cell clones over time is indicated by an increase in D. The lower the D value, the less diverse the population.

The state of the cell’s sequential and efficient immune responses was evaluated with C-ImmSim. Immune cell memory causes a small number of cells to have a significantly longer half-life than other cells. The immune simulation conducted by the ImmSim server showed consistency in real-life reactions. Elevated levels of IgM demonstrated a swift primary response. Also, there was a direct relationship between the growth of the B cell population and immunoglobulin expression (IgG1 + IgG2, IgM, and IgG + IgM), leading to the decrease in the concentration of the antigen (Figures 9A,B). As memory developed, the number of Th cells and cytotoxic T (CT) cells increased significantly (Figures 10A,B). IFN-γ production was also found to be stimulated after immunization (Figure 10C). As the memory developed, the T cell population outcomes became significantly approachable, and all other immune cell populations were exposed to be consistent.

## Discussion

TB is a serious and deadly infection, while BCG is the only available anti-TB vaccine. However, the BCG does not provide sufficient protection for TB-infected persons (Riccomi et al., 2019). As a result, there is a pressing need for new TB vaccine candidates. There are several vaccine candidates currently undergoing clinical trials. In recent years, reverse vaccinology coupled with genomics and proteomics data availability has aided vaccine development. Furthermore, the implementation of efficient bioinformatics tools has been proven more advantageous (Reginald et al., 2018).

Immunogenic antigen identification is critical for vaccine development for the *in silico* prediction of epitopes (Bahrami et al., 2019). The antigens bind to immune cell receptors or ligands to initiate an immune response (Galluzzi et al., 2017). As a result, we used computational methods to comprehensively evaluate three Mtb antigens (Rv0058, Rv0101, and Rv3343) using bioinformatics methods. Cellular and humoral immunity inducible B and T cell Mtb epitopes were predicted using bioinformatics (Yazdani et al., 2020). These epitopes were selected for vaccine prediction due to their critical role in cellular and biological growth. The prediction of B-cell epitopes is another crucial aspect of vaccine development (Dubey et al., 2018), as it provides interphase for antigen–antibody binding (El-Manzalawy et al., 2008; Lu et al., 2018; Krocova et al., 2020).

Epitopes of T-cells are required for immune stimulation and the cooperation of MHC molecules (Sunita et al., 2020). As a result, choosing MHC-fixing epitopes is critical for predicting potent T-cell epitopes (Eickhoff et al., 2019). Also, recognizing the CD4^+^ and CD8^+^ T cells is essential in the development of MSV (Russell et al., 2019; Patankar et al., 2020). B- and T-cell epitopes were predicted from selected antigens linked with AAY and GPGPG (Meza et al., 2017). These two linkers aided in generating a reduced-junctional immunogenic sequence allowable for the MSV model (Meza et al., 2017). The activity and expression of the vaccine were improved by fusing the EAAAK linker (Arai et al., 2001) to connect the adjuvant and the epitope. We identified several other MHC, IFN, and B-cell epitopes when evaluating the vaccines informatics. IFN-γ has been reported to antagonize TB in the lungs of mice (Sakai et al., 2016). The absence of an allergenic component has boosted the protein vaccine’s effectiveness as a candidate vaccine. On Vaxijen v2.0 servers, antigenicity scores for the built MSV were higher. MSV require adjuvants due to their lower immunogenicity.

The final protein was highly soluble and immunogenic of 34.1 kDa size. The protein’s overexpression function in *E. coli* was investigated (Khatoon et al., 2017). The vaccine had a theoretical pI of 9.5, indicative of its basicity—the proteins expected instability index score of 29.94 indicated high stability when expressed. The aliphatic index determines whether a protein has aliphatic side chains, indicating hydrophilicity. The secondary and tertiary protein structure is critical for developing vaccines (Meza et al., 2017). The study revealed 50% coils for the protein’s secondary structure. The coils conform to the natural antigenicity of native unfolded secondary proteins, mostly in α-helices. Upon folding into its native state, the spiral structure is recognized by antibodies in immune response (Corradin et al., 2007). According to the Ramachandran plot’s findings, the vaccine’s 3D model was significantly enhanced when refined and showed acceptable features. The Ramachandran plot indicates a 95.49% residue start at preferred domains, while only 4.6% in the allowed regions indicates a satisfactory overall model output. The validity of a vaccine candidate should meet the immuno-reactive prerequisite in a serological sample (Gori et al., 2013).

Furthermore, the association of TLR-3 with TLR on immune cells was evaluated using the MD simulation system through the vaccine-TLR-3 docking complex. The RMSD plot showed a stable binding of the complex. Our study showed that TLR-3 had a high affinity for the developed vaccine. TLR-3 interaction with the vaccine suggested that it could elicit both innate and adaptive inflammatory responses (Crozat and Beutler, 2004).

Immune simulations produced results that matched those observed in real-life inflammation responses. The result generally showed a rise in inflammatory responses after being repeatedly exposed to antigen. B and T cells developed, with prolonged B-cell memory over months. Th cells constituted the majority of the cells elicited. A significant discovery showed that IFN and IL-2 levels rose after the first injection with a persisted stability after multiple antigen exposures. This indicates a large number of Th cells and, as a result, efficient immunoglobulin production, suggesting a humoral response. Clonal specificity inquiry, Simpson index, D, indicates a potential immune response of higher diversity.

These diverse characteristics necessitate gene expression in an *E. coli* host (Chen, 2012; Rosano and Ceccarelli, 2014). The codon of the first six amino acids was optimized to enable *E. coli* to express a high amount of the vaccine protein. The GC content of 59.48% and CAI score (0.968) were suitable for bacterial expression systems. Subsequent expression of the peptide in the bacterial system coupled with some analysis may confirm the reports of the immuno-informatics research.

## Conclusion

The computational methods described in this article may provide new information about Mtb vaccine antigens that could not be obtained through preclinical, *in vitro*, or animal studies alone. This study utilized an immuno-informatics route to describe a novel TB-MSV with potential immunogenicity that satisfies the requirement for developing a vaccine. The B- and T-cell epitopes evaluated under this study showed immunogenic improvement after fusing a suitable linker and an adjuvant. The vaccine’s antigen and allergen characteristics met the standard requirement for vaccine development. The solubility, physicochemical properties, and structural analyses also were of excellent quality. Molecular docking and simulations of TLR-3 and vaccines were used to measure the complex’s avidity. *In silico* modulation was used to validate the antigenic clearance against immune cells. The codon optimization also yielded a positive CAI value, which could help further *in vivo* expression. This study used several immune-informatics tools to investigate the varying properties of vaccines. Furthermore, the expected epitopes-based subunit vaccine evaluation is perfectly acceptable for demonstrating immunogenicity and potential as a TB vaccine candidate. The current findings in this study will be validated further *via in vivo* experimental investigation in the future.

## Data Availability Statement

The original contributions presented in the study are included in the article/supplementary files, further inquiries can be directed to the corresponding authors.

## Author Contributions

TZ supervised the project and was responsible for the funding acquisition for this study. TZ, HMAH, and ZK conceptualized and designed the study. ZK performed the immuno-informatics. ZK and DU wrote the manuscript. TZ and HMAH critically revised and edited the manuscript. DU, OA, SS, and HMAH helped in verifying and discussing the results. All authors contributed to the article and approved the submitted version.

## Funding

This work was supported by the National Key R&D Program of China (2021YFA1300904), “One Belt One Road” (to ZK) master fellowship program for international students, and China postdoctoral fellowship, Guangdong province, Huangpu, and GIBH-University of Chinese Academy of Sciences (to HMAH). The funders had no role in the study design, data collection, and analysis, decision to publish, or preparation of the manuscript.

## Conflict of Interest

The authors declare that the research was conducted in the absence of any commercial or financial relationships that could be construed as a potential conflict of interest.

## Publisher’s Note

All claims expressed in this article are solely those of the authors and do not necessarily represent those of their affiliated organizations, or those of the publisher, the editors and the reviewers. Any product that may be evaluated in this article, or claim that may be made by its manufacturer, is not guaranteed or endorsed by the publisher.

## Footnotes

^1^ https://www.uniprot.org

^2^ https://uniprot.org

^3^ http://www.iedb.org/

^4^ https://www.cbs.dtu.dk/services/NetCTL/

^5^ http://crdd.osdd.net/raghava/ifnepitope/

^6^ http://www.imtech.res.in/raghava/abcpred/

^7^ http://www.ddg-pharmfac.net/Vaxijen/VaxiJen/VaxiJen.html

^8^ http://crdd.osdd.net/raghava/algpred/

^9^ https://webs.iiitd.edu.in/raghava/toxinpred/multisubmit.php

^10^ http://aps.unmc.edu/AP/main.php

^11^ https://web.expasy.org/protparam/

^12^ http://protein-sol.manchester.ac.uk

^13^ http://bioinf.cs.ucl.ac.uk/psipred/

^14^ https://npsa.lyon.inserm.fr/cgi-bin/npsa_automat.pl?page=/NPSA/npsa_sopma.html

^15^ http://robetta.bakerlab.org

^16^ http://galaxy.seoklab.org/cgi-bin/submit.cgi?type=REFINE

^17^ https://dokhlab.med.psu.edu/chiron/login.php

^18^ https://prosa.services.came.sbg.ac.at/prosa.php

^19^ http://services.mbi.ucla.edu/ERRAT/

^20^ http://mordred.bioc.cam.ac.uk/rapper/rampage.php

^21^ http://tools.iedb.org/ellipro/

^22^ https://www.rcsb.org

^23^ http://cadd.zju.edu.cn/hawkdock/

^24^ http://bioinfo3d.cs.tau.ac.il/FireDock/php.php

^25^ http://imods.Chaconlab.org/

^26^ http://www.prodoric.de/JCat

^27^ http://150.146.2.1/C-IMMSIM/index.php
